# Characterization of Novel Lytic *Myoviridae* Phage Infecting Multidrug-Resistant *Acinetobacter baumannii* and Synergistic Antimicrobial Efficacy between Phage and Sacha Inchi Oil

**DOI:** 10.3390/ph15030291

**Published:** 2022-02-26

**Authors:** Phitchayapak Wintachai, Supayang Piyawan Voravuthikunchai

**Affiliations:** 1 School of Science, Walailak University, Nakhon Si Thammarat 80161, Thailand; 2 Center of Antimicrobial Biomaterial Innovation-Southeast Asia and Natural Product Research Center of Excellence, Faculty of Science, Prince of Songkla University, Songkhla 90110, Thailand; supayang.v@psu.ac.th

**Keywords:** *Myoviridae* phage, *Acinetobacter baumannii*, bacteriophage, antibacterial activity, biofilms, sacha inchi oil, synergistic activity, phage therapy

## Abstract

Multidrug-resistant (MDR) strains of *Acinetobacter baumannii* have become a major cause of hospital-acquired infections, resulting in an increase in morbidity and mortality worldwide. Many alternative treatments, including phage therapy, are attractive approaches for overcoming problems posed by antibiotic resistance. A newly isolated phage, vWUPSU-specific MDR *A. baumannii*, showed a narrow host range against MDR *A. baumannii*. This research was conducted to isolate, characterize, and apply the phage with sacha inchi oil as an alternative antimicrobial agent. Genome analysis suggested that phage vWUPSU is a novel phage belonging to the family *Myoviridae*, order *Caudoviridae*. This phage prevented biofilm formation and eradicated preformed biofilms in a dose-dependent manner. In addition, a synergistic antimicrobial effect of the interaction between phage vWUPSU and sacha inchi oil on planktonic cells was observed. The combination of phage and sacha inchi oil significantly inhibited and removed biofilms, compared with the effects of either single treatment. The results of this work indicate that phage vWUPSU could potentially be applied to control MDR *A. baumannii.* The antibacterial and antibiofilm activities of the combination of phage vWUPSU and sacha inchi oil have attracted significant interests in the development of antibacterial phage products as beneficial treatment options.

## 1. Introduction

Multidrug-resistant *Acinetobacter baumannii* (MDR *A. baumannii*), a Gram-negative pathogenic bacterium, is an important causative agent of nosocomial and community-acquired infections. MDR *A. baumannii* causes a wide range of infections, including bloodstream infections, osteomyelitis, pneumonia, urinary tract infections, and wound infections [1]. MDR *A. baumannii* is becoming more challenging to control and treat, resulting in increased risks in morbidity, prolonged lengths of hospital stay, prolonged lengths of ICU stay, and mortality [2]. Recently, the World Health Organization (WHO) listed *A. baumannii* as a critical priority of the ESKAPE Pathogen Program, which focuses on the urgent need for new treatments [3]. The ability of *A. baumannii* to colonize and form biofilms on inert or living surfaces has been reported. Biofilms, which constitute the important virulence factor contributing to chronic and persistence infections of *A. baumannii*, are populations of bacterial cells encased in a matrix of extracellular polymeric secretions (EPS). Biofilms function as a physical barrier to antimicrobial penetration that can reduce the diffusion of antimicrobial agents, which contributes to the antimicrobial tolerance of biofilms. Moreover, biofilm matrix can protect bacterial cells from host immune defense, rendering bacterial biofilms extremely resistant to eradication.

Bacteriophages, or phages, are an abundant biological entity and are prokaryotic viruses that infect specific bacteria. Over the last decades, phages have been reported to be effective at treating various infections, such as burn wound sepsis [4], lung infections [5,6], and lung infections specific to patients with cystic fibrosis [7]. Due to increasing bacterial resistance to antibiotics and the specificity of phage and host interactions, there has been renewed interests in using phages as either alternatives or supplements to antibiotics to combat MDR bacteria [8,9]. In recent years, the antibacterial and antibiofilm activities of several phages targeting MDR *A. baumannii* have been characterized [10,11]. For example, phage ΦAB1- and phage φAbp2-specific MDR *A. baumannii* were characterized, and genomic analysis was performed [12,13,14]. Phage ΦAB1 is a member of the subfamily *Autographivirinae* of the family *Podoviridae*, and phage φAbp2 belongs to the subfamily *Peduovirinae* of the family *Myoviridae.* Phage ISTD, which is active against carbapenem-resistant *A. baumannii*, inhibited both planktonic and biofilm-associated viable bacterial cells [15]. Phage IsfAB78 displayed antibiofilm activity against colistin-resistant *A. baumanni* [16]. Phage Bϕ-R2096 showed high efficacy in *Galleria mellonella* larvae and mouse model treatments [17]. Phage Abp1 eliminated pan-drug-resistant *A. baumannii* infections in HeLa cells and the mouse model [18]. Interestingly, a phage therapy clinical trial involving patients who developed MDR *A. baumannii* craniectomy site infections was performed [19]. Bacteriophages were administered intravenously. *A. baumannii* infection at the craniotomy site was removed, and recoveries of the craniotomy site and skin flap were reported. The administration of bacteriophages cocktails to treat a patient with an MDR *A. baumannii* infection has been reported. The clearance of MDR *A. baumannii* infection was detected, and the patient recovered [20]. The results shed light on an option to treat multidrug-resistant bacterial infections.

Additionally, natural antibacterial agents such as oils, essential oils, and plant derivatives are recognized as offering powerful approaches to the treatment of bacterial infections. Therefore, natural products may serve as alternative treatments in synergy with phages to control MDR *A. baumannii* infections. Natural products could be used as additives in phage treatment medications. Sacha inchi oil or Inca inchi oil is extracted by pressing the seeds of *Plukenetia volubilis* Linneo, which belongs to the *Euphorbiaceae* family. This plant is native to Peruvian Amazon regions but currently is commercially cultivated around the world. Sacha inchi oil is traditionally used in Peru for skin care because of its moisturizing and anti-irritation potential [21]. Sacha inchi oil also exhibits antibacterial and anti-inflammation activities [22,23]. Growing interest in the benefits of sacha inchi oil is fostering the development of cosmetics and skincare products such as the formulations of sacha inchi oil emulsions [24]. However, there has been no report published regarding the combined application of sacha inchi oil and phages to inhibit specific bacterial infections.

In this study, a novel phage, vWUPSU-infecting *A. baumannii*, was isolated. Biological characterization and genomic analysis of the phage were conducted. Various combinations of phage vWUPSU and sacha inchi oil were used, and antibacterial and antibiofilm activities were assessed to determine the potential for developing therapeutic agents for external use in combatting infections.

## 2. Results

### 2.1. Phage Isolation and Purification

**Figure 1:**
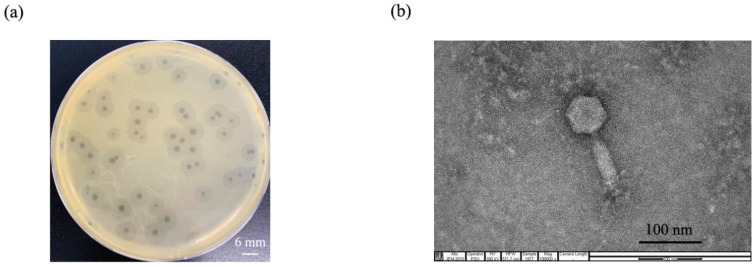
Phage isolation and morphological characterization of the phage particle. (**a**) Morphology of phage vWUPSU plaques. The conventional double-layer agar method was performed to observe plaque morphology. MDR *A. baumannii* NPRCOE 160519 was used as the host strain. The plaques were observed and photographed after incubation at 37 °C overnight. The scale bar represents 6 mm; (**b**) phage morphology under transmission electron microscope (TEM). Phage particles were stained by a negative staining technique and visualized under JEOL JEM-2010 TEM at an acceleration voltage of 160 kV (100,000× magnification). The scale bar represents 100 nm.

A phage was enriched from hospital wastewater samples using MDR *A.baumannii* clinical isolate NPRCOE 160519 as the host bacterial strain. After it was purified three times, the phage produced plaques 3–6 mm in diameter surrounded by halos on an MDR *A. baumannii* lawn (Figure 1a).

### 2.2. Phage Morphology

The morphology of phage particles was observed under transmission electron microscope (TEM). The phage contained a hexagonal head with a diameter of 60.19 ± 3.36 nm, a contractile tail with a length of 92.59 ± 6.41 nm, and a baseplate at the tip of the tail (*n* = 3) (Figure 1b). Following the current guidelines of the International Committee on Taxonomy of Viruses (ICTV), phage vWUPSU morphology indicated that it is classified in the order *Caudovirales*, family *Myoviridae*, a family of contractile tail double-stranded DNA viruses. The phage was named based on phage morphology and host as *Acinetobacter* phage vB_AbM_WUPSU (phage vWUPSU).

### 2.3. Host Range Analysis and Efficacy of Plating (EOP)

The host range of phage vWUPSU was assessed using 30 clinical MDR *A. baumannii* isolates, *Escherichia coli*, *Klebsiella pneumoniae*, Methicillin-resistant *Staphylococcus aureus* (MRSA), and *Pseudomonas aeruginosa*. Phage vWUPSU lysed 16 clinical MDR *A. baumannii* isolates (53.3%). The phage did not show any lytic activity against *E. coli*, *K. pneumoniae*, MRSA, or *P. aeruginosa* (Table 1). To further evaluate the infection of phage vWUPSU, EOP analysis was conducted, and the EOP value, a ratio of lysis plaques produced on the bacterial lawn of each susceptible strain divided by the number of plaques produced on the bacterial lawn of the host strain, was calculated. High, moderate, and low production was found for seven, six, and three isolates, respectively. No strains showed a higher production value than the host strain.

### 2.4. Whole-Genome Analysis and Annotation

**Figure 2:**
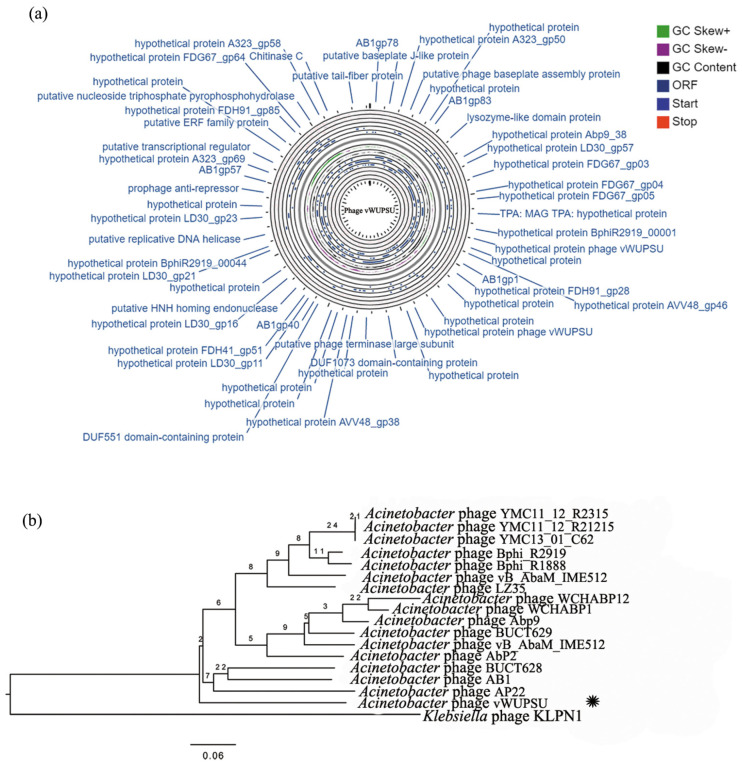

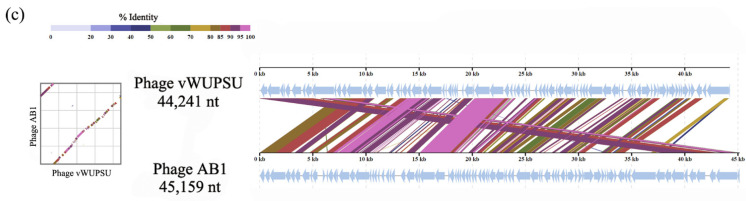
Phage vWUPSU genomic analysis. (**a**) Map of phage vWUPSU genome. The genome map was generated by GC viewer server [28]; (**b**) phylogenetic tree of phage vWUPSU constructed based on the complete genome sequences of selected phages in NCBI BlastN [29]. The phylogenetic tree was produced by VICTOR Classification and Tree Building Online Resource server [30]. The query sequence is marked with an asterisk. (**c**) The whole genome sequence alignment of phage vWUPSU with phage AB1 that had the highest similarity to phage vWUPSU. Homologous regions detected by a TBLASTX search were connected by segments colored based on amino acid identity [29]. The alignment was generated by TBLASTX using ViPTree server [27]. The color bar shows the % identity of TBLASTX.

The whole genome sequences of phage vWUPSU were characterized using the Illumina platform and then annotated. Phage vWUPSU has a double-stranded DNA with a length of 44,241 base pairs (Figure 2a). The GC content of the phage genome was 37.2%. The genome contained 83 putative open reading frames (ORFs): 70 ORFs on the negative stand and 13 ORFs on the positive strand (Supplementary Table S1). A total of 75 ORFs started with start codon ATG, while seven ORFs had start codon GTG. One ORFs had start codon TTG. There were no tRNA or antibiotic resistance genes in the phage genome. The functions of the ORFs in the genome were predicted by searching using BLASTX against the NCBI database. Eighty-three predicted genes encoded 83 predicted proteins: 21 were known putative functional proteins and 62 were hypothetical proteins. The largest and smallest ORFs were ORF75 and ORF19, which encoded a putative tail fiber protein with 693 amino acids and a hypothetical protein of phage vWUPSU with 116 amino acids, respectively. With regard to the putative functional proteins, 18 and 3 predicted proteins were situated on the negative and positive strands, respectively. The predicted functional proteins were classified into four types: four phage structure-related proteins (putative baseplate J-like protein, putative phage baseplate assembly protein, putative tail fiber-lysozyme protein, and putative tail-fiber protein), seven proteins involved in DNA replication/modification (AB1gp78, putative phage terminase large subunit, putative HNH homing endonuclease, putative replicative DNA helicase, putative transcriptional regulator, putative ERF family protein, and putative nucleoside triphosphate pyrophosphohydrolase), three proteins related to host lysis (lysozyme-like domain, chitinase C, and phage holin family proteins), and seven predicted proteins with additional functions (AB1gp83, AB1gp1, DUF1073 domain-containing protein, DUF551 domain-containing protein, AB1gp40, prophage-anti repressor, and AB1gp57). Five ORFs on the positive strand were not matched with the proteins in the databases. They were classified as hypothetical proteins of phage vWUPSU. The genome of phage vWUPSU was also annotated by the RAST server (Supplementary Table S2) [26]. Phage genome genes were predicted to be of five groups: phage structural related proteins (capsid maturation protease, tail fiber protein, and tail length tape-measure protein), proteins involved in DNA replication/modification (single-stranded-DNA-specific exonuclease, putative replicative DNA helicase, DNA helicase, terminase large subunit, terminase small subunit, putative single-stranded DNA binding protein, deoxynucleoside kinase, and methyltransferase type 11), proteins related host lysis (endolysin, holin, and phage holin), and hypothetical proteins.

The similarity of phage vWUPSU with other phages in the NCBI database was comparatively analyzed by BLASTN. The relationships of phage vWUPSU with other related phages were analyzed using a phylogenetic tree. Phage vWUPSU was grouped in the same clade as *Acinetobacter* phage AB1 (NC_042028.1) and *Acinetobacter* phage BUCT628 (MZ593728.1). The genome of phage vWUPSU showed the highest similarity at the DNA level to those of phage AB1 and phage BUCT628, with 91.9% and 91.3% identity, respectively (Figure 2b). To determine the homologous region on the genome of phage vWUPSU and the highest similarity phage, phage AB1, genome sequence comparisons of phage vWUPSU and phage AB1 were performed with TBLASTX using the ViPTree server [27]. Some regions on the genome of phage vWUPSU showed more than 50% identity with the genome of *Acinetobacter* phage AB, while other regions on the genome showed less than 50% identity (Figure 2c). The analysis showed that contig 35 of phage vWUPSU showed the highest identity with AB1gp30 of phage AB1 (YP_009613795, 96.7% identity). Contig 36 had 98.7% similarity with AB1gp31 of phage AB1 (YP_009613796). Moreover, contig 22 and contig 23 displayed high similarity with AB1gp13 of phages AB1 (YP_009613778, 95.7% identity) and AB1gp15 (YP_009613780, 99.4% identity), respectively. Phage vWUPSU was categorized into the order *Caudovirales*, *Myoviridae* family, and genus *Obolenskvirus*. The percent identity of phage vWUPSU and phage AB1 is less than 95%, indicating that phage vWUPSU is a new species.

**Figure 3:**
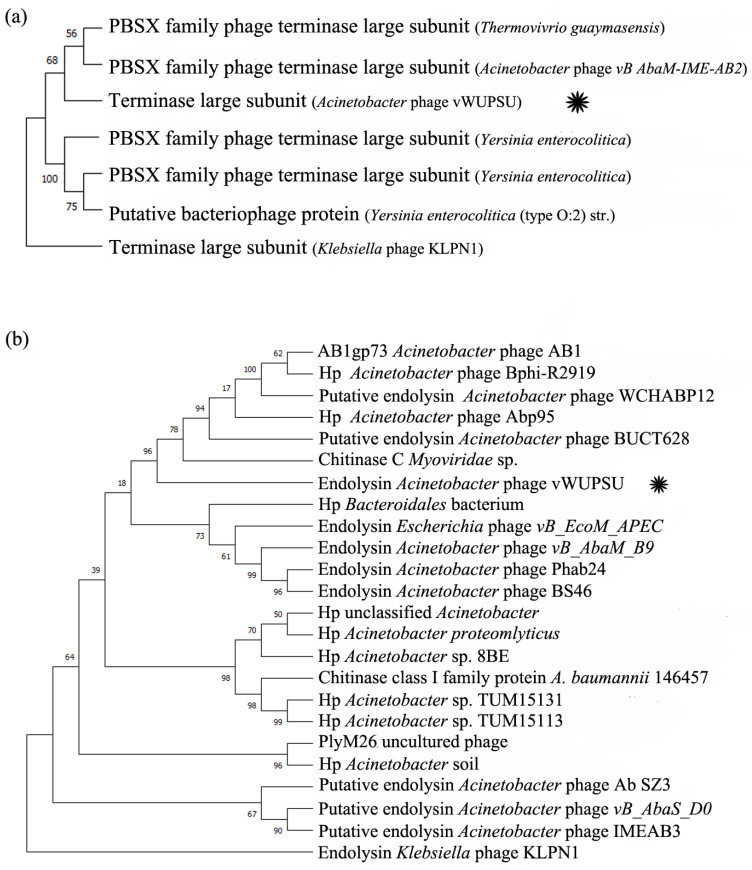
Phylogenetic tree of phage terminase large subunit and endolysin. The maximum likelihood phylogenetic trees of (**a**) phage terminase large subunit and (**b**) endolysin based on the alignment of amino acid sequences were constructed by maximum-likelihood phylogenetic tree based on the JTT matrix-based model using 1000 bootstrap replicates [33]. The query sequence is marked with an asterisk. Hp is a hypothetical protein.

The phylogenetic relationships between phage vWUPSU and its relatives were also evaluated by constructing the phylogenetic trees using a comparison of the individual genes. Phage terminase large subunit and endolysin were selected for analysis. The phage terminase large subunit of phage vWUPSU was clustered together with the PBSX family terminase large subunit of *Acinetobacter* phage vB_AbaM-IME-AB2 (YP_006383827.1) and the PBSX family phage terminase large subunit of phage *Thermovibrio guaymasensis* (WP_211321837.1) with a bootstrap value of 68% (Figure 3a). Phage terminase large subunit of *Klebsiella* phage KLPN1 (AKS10661.1) was selected as an outgroup. To further understand the genetic relationships of the genes, phylogenetic analysis of phage endolysin with its relatives was performed. Phage endolysin, a diverse class of enzymes, is a phage-encoded peptidoglycan hydrolase that plays a key role in the degradation of the host cell wall [31,32]. The endolysin sequence of phage vWUPSU was grouped into the same clade as *Acinetobacter* phages, and the endolysin of phage vWUPSU was closely related to TPA: MAG TPA: chitinase C of *Myoviridae* phage (DAF69039.1) with a bootstrap value of 96% (Figure 3b). Endolysin of *Klebsiella* phage KLPN1 (AKS10699.1) was selected as an outgroup. The results of phylogenetic tree analysis indicated that phage vWUPSU was a new species of virus that belongs to *Obolenskvirus*, family *Myoviridae*, and order *Caudovirales*.

### 2.5. Analysis of the Phage Adsorption Rate and One-Step Growth Curve

**Figure 4:**
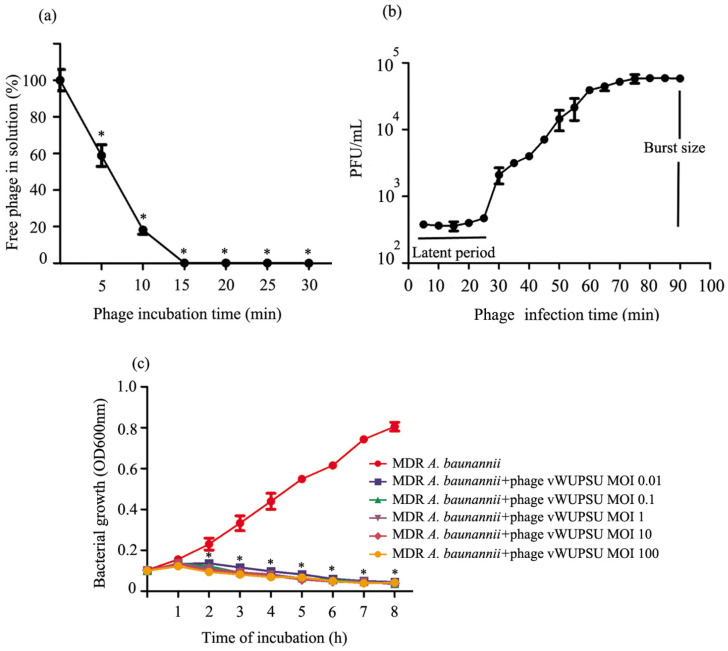
Biological characterization of phage vWUPSU. (**a**) Adsorption rate of phage vWUPSU. The phage was mixed with MDR *A. baumannii*, and the nonadsorbed infectious phages were serially counted by a double-layer agar method. (**b**) One step growth curve of phage vWUPSU. (**c**) Kinetics of lytic development of phage vWUPSU in MDR *A. baumannii*. Bacterial culture density was measured at OD600 every hour for 8 h. Experiments were undertaken independently in duplicate with duplicate assay. The data show mean ± SD (*, *p* value < 0.05).

The adsorption rate of phage vWUPSU onto MDR *A. baumannii* was investigated. The number of free phages in solution was measured every 5 min for 30 min by standard plaque assay. The results showed that more than 80% of the phage particles were adsorbed within 10 min (Figure 4a). The one-step growth curve of phage vWUPSU was analyzed. The latent period of phage vWUPSU, a period between the absorption of phage vWUPSU to bacterial cells and the beginning of the lysis of bacterial cells, was approximately 25 min. The burst size, the final concentration of phage vWUPSU, and the concentration of host bacterium was 153 plaque forming units (PFU) per infected cell (Figure 4b).

### 2.6. Lytic Activity

The lysis kinetics of phage vWUPSU were measured every hour by a killing curve. The absorbance of uninfected bacterial culture, a control, increased rapidly, but the absorbance of phage vWUPSU-infected MDR *A. baumannii* at multiplicity of infection (MOIs) of 0.01, 0.1, 1, 10, and 100 significantly decreased at 2 h post-infection (*p* ≤ 0.04). At 8 h post-infection, the absorbance of bacterial cells was nearly completely reduced (Figure 4c).

### 2.7. Transmission Electron Microscopy (TEM) of Phage Infected Bacteria

**Figure 5:**
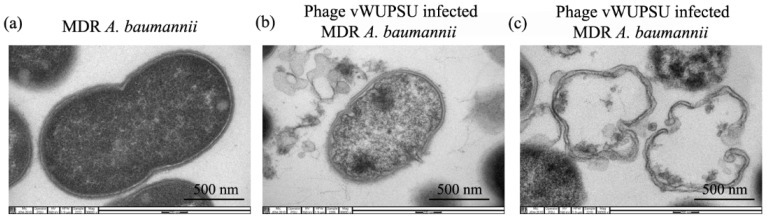
Bacterial cell ultrastructural morphology under TEM. (**a**) MDR *A. baumannii*; (**b**,**c**) phage vWUPSU infected-MDR *A. baumannii* was observed under a JEOL JEM-2010 transmission electron microscope at an acceleration voltage of 160 kV (30,000× magnification). The scale bar represents 500 nm.

The ultrastructural changes in MDR *A. baumannii* infected with phage vWUPSU were observed by TEM. Uninfected MDR *A. baumannii* cells displayed normal morphological characteristics with intact cell membranes and uniform cytoplasmic density (Figure 5a). Morphological changes in MDR *A. baumannii* cells after phage treatment were detected. The phage induced pits on the bacterial cell walls, and phage infected cells displayed the leakage of cellular contents (Figure 5b). Membrane damage, bacterial cell death, and lysis were observed (Figure 5c).

### 2.8. Thermal and pH Stability Test

**Figure 6:**
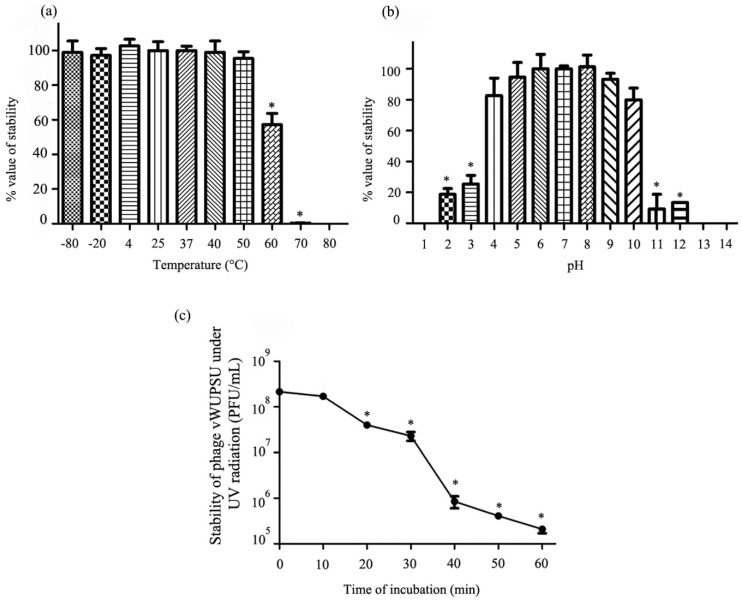
Stability of phage vWUPSU under varying conditions. (**a**) Effect of varying temperature on phage vWUPSU stability; (**b**) stability of phages at varying pH at 37 °C; (**c**) stability of phage under UV radiation. Experiments were undertaken independently in duplicate with duplicate assay. The data show mean ± SD (*, *p* value < 0.05).

The stability of phage vWUPSU under various temperatures and pH values was evaluated. The thermal stability of the phage was determined after incubation at 4 to 80 °C for 2 h. Phage stability after freezing at −80 to −20 °C for 2 h and thawing was also evaluated. There were no significant changes in phage vWUPSU viability between −80 and 50 °C, but a significant reduction was observed at 60 °C and 70 °C (*p* ≤ 0.02). The phage completely lost viability after incubation at 80 °C (Figure 6a). For phage stability at different pH values, phage vWUPSU was incubated in SM buffers of different pH values ranging from 1 to 14 for 2 h. The phage showed no significant variation in stability after incubation at pH 4 to 10. Incubation at pH 2, 3, 11, and 12 significantly reduced phage viability (*p* ≤ 0.003). The phage completely lost viability after incubation at pH 1, 13, and 14 (Figure 6b).

### 2.9. UV Stability Test

The stability of phage vWUPSU after UV light exposure was assessed. A significant reduction of approximately 60% in phage stability was observed at 20 min post-UV exposure, and phage stability was significantly reduced to less than 0.1% at 60 min post-UV exposure (*p* ≤ 0.001). (Figure 6c).

### 2.10. Antibiofilm Activity of Phage vWUPSU

**Figure 7:**
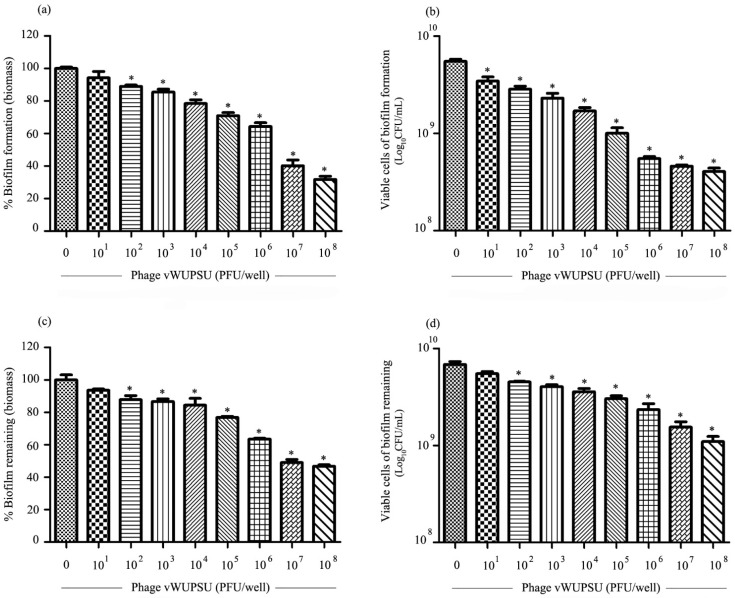
Antibiofilm effect of phage vWUPSU. To determine the activity of phage vWUPSU with regard to preventing biofilm formation, MDR *A. baumannii* was added to the 96-well plates and then supplemented with phage vWUPSU. The mixture was incubated at 37 °C without agitation for 24 h. The biofilm of MDR *A. baumannii* filled with broth medium (TSB + SM buffer) was used as a negative control. (**a**) The effect of phage vWUPSU treatment on biofilm formation was assessed by biomass evaluation. (**b**) The effect of phage vWUPSU treatment on biofilm cell viability was quantitated. To determine the phage activity with regard to the removal of preformed biofilms, MDR *A. baumannii* was cultured at 37 °C overnight. The biofilm was incubated with phage vWUPSU for 24 h. The preformed biofilm filled with broth medium (TSB + SM buffer) was considered as a negative control. (**c**) The effect of phage vWUPSU treatment on preformed biofilm was assessed by biomass evaluation. (**d**) The effect of phage vWUPSU treatment on biofilm cell viability was quantitated. For testing the activity of phage vWUPSU against biofilm formation and preformed biofilms, the numbers of phage vWUPSU at an MOI of 1 were 1 × 10^7^ PFU/well and 1 × 10^8^ PFU/well, respectively. Experiments were undertaken independently in triplicate with duplicate assay. The data show mean ± SD (*, *p* value < 0.05).

The ability of phage vWUPSU to inhibit biofilm formation and remove preformed biofilms was assessed by measuring biofilm biomass and cell viability. With regard to its biofilm inhibitory efficacy, phage vWUPSU at 1 × 10^2^ to 1 × 10^8^ PFU/well significantly inhibited biofilm biomass by approximately 5.8–68.3% relative to the control (*p* ≤ 0.01) (Figure 7a). Cell viability was significantly reduced by 0.2–1.5 log after treatment with phage vWUPSU at 1 × 10^1^ to 1 × 10^8^ PFU/well (*p* ≤ 0.02) (Figure 7b). The efficacy of phage vWUPSU with regard to the inactivation of preformed biofilms was also evaluated. Significant reductions of approximately 6.4–53.3% in biofilm biomass were observed after treatment with phage vWUPSU at 1 × 10^2^ to 1 × 10^8^ PFU/well (*p* ≤ 0.048) (Figure 7c). Bacterial cell viability significantly decreased by 0.1–0.6 log (*p* ≤ 0.03) (Figure 7d). The results demonstrated that phage vWUPSU decreased both biofilm formation and preformed biofilms in a dose-dependent manner.

### 2.11. Phage Sensitivity to Sacha Inchi Oil

**Figure 8:**
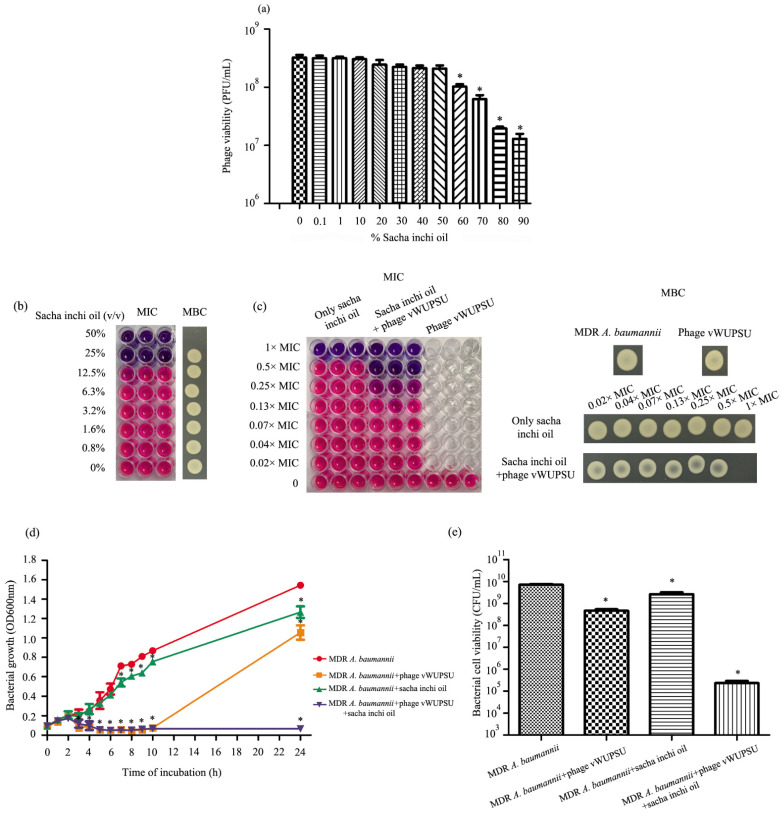
Possibility of the combination of phage vWUPSU and sacha inchi oil. (**a**) Phage viability after incubation with sacha inchi oil at 0.1% (*v*/*v*) to 90% (*v*/*v*). (**b**) Minimum inhibitory concentration (MIC) and minimal bactericidal concentration (MBC) values of sacha inchi oil. (**c**) MIC and MBC of the combination of phage vWUPSU at an MOI of 1 and sacha inchi oil. The lowest concentration before the color change was selected to determine the MIC. The concentration at which bacteria did not grow was the MBC value. (**d**) Lytic effect of a combination of sacha inchi oil at 0.5× MIC and phage vWUPSU at an MOI of 1 in vitro. (**e**) Impact of a combination of sacha inchi oil at 0.5× MIC and phage vWUPSU at an MOI of 1 on viable cell numbers. Experiments were undertaken independently in duplicate with a duplicate assay. The data show mean ± SD (*, *p* value < 0.05).

The stability of phages vWUPSU in sacha inchi oil was assessed by incubating the phage with varying concentrations of sacha inchi oil (0.1–90%). At 24 h post-incubation, there was no significant effect of sacha inchi oil at concentrations of 0.1–50% on phage vWUPSU viability. A significant reduction in phage viability was observed after incubation with sacha inchi oil at concentrations of 60–90% (*p* ≤ 0.01) (Figure 8a).

### 2.12. Combined Application of Sacha Inchi Oil and Phage

Sacha inchi oil was evaluated for antimicrobial activity against clinically isolated MDR *A. baumannii*. The minimum inhibitory concentration (MIC) and minimal bactericidal concentration (MBC) values of sacha inchi oil were 25% (*v*/*v*) and 50% (*v*/*v*), respectively (Figure 8b). The combination of phage vWUPSU at an MOI of 1 and sacha inchi oil had MIC and MBC values of 12.5% (*v*/*v*) and 25% (*v*/*v*), respectively (Figure 8c). The effect of phage vWUPSU in combination with sacha inchi oil was determined by the fractional inhibitory concentration (FIC) index. The results showed an FIC index at 0.5, indicating a synergistic effect between phage vWUPSU and sacha inchi oil [34].

### 2.13. Bacteriolytic Activity of the Combination of Phage vWUPSU and Sacha Inchi Oil

The bacteriolytic activity of the combination of phage vWUPSU and sacha inchi oil was evaluated. Bacterial growth and bacterial cell viability were evaluated compared to phage vWUPSU alone, sacha inchi oil alone, and no treatment. In the treatment with only phage vWUPSU at an MOI of 1, phage vWUPSU significantly inhibited the growth of MDR *A. baumannii* during the first 10 h (*p* < 0.0001), but the turbidity of the MDR *A. baumannii* culture increased at 24 h post-incubation (Figure 8d). Phage vWUPSU at an MOI of 1 reduced bacterial cell viability by 1.25 log (Figure 8e). Sacha inchi oil at 0.5× MIC reduced bacterial growth at 24 h post-incubation (Figure 8d). Bacterial cell viability decreased by approximately 0.45 log when compared with untreated MDR *A. baumannii* (Figure 8e). Treatment with the combination of phage vWUPSU at an MOI of 1 and sacha inchi oil at 0.5× MIC significantly inhibited the growth of the bacteria at 4 h post-treatment (*p* = 0.01) (Figure 8d). Absorbance and cell viability were also assessed at 24 h post-incubation. The results showed that the combination of phage vWUPSU at an MOI of 1 and sacha inchi oil at 0.5× MIC significantly inhibited bacterial growth (*p* < 0.0001). For viable cells, the combination of phage vWUPSU and sacha inchi oil at 0.5× MIC significantly decreased the number of viable cells, approximately 4.49 log at 24 h post-incubation compared with only phage or only sacha inchi oil treatments (*p* ≤ 0.001) (Figure 8e). The results showed synergistic activities of phage vWUPSU and sacha inchi oil.

### 2.14. Evaluation of the Efficacy of the Combined vWUPSU and Sacha Inchi Oil under SEM

**Figure 9:**
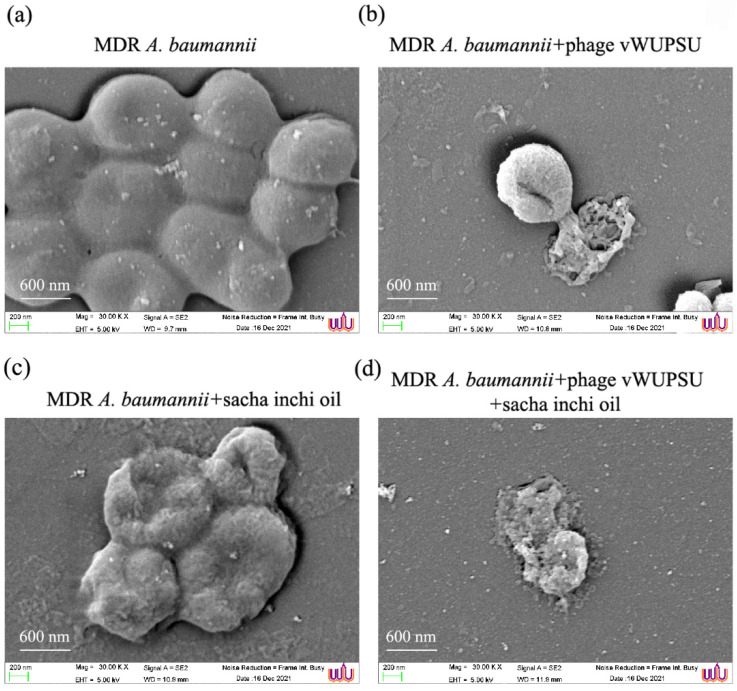
Ultrastructural analysis of MDR *A. baumannii* cells after treatments. MDR *A. baumannii* cells were incubated with (**a**) only TSB, (**b**) phage vWUPSU at an MOI of 1, (**c**) sacha inchi oil at 0.5× MIC, and (**d**) the combined phage vWUPSU and sacha inchi oil for 3 h. Then, the cells were collected, fixed, dehydrated, and observed under a field emission scanning electron microscope at an acceleration voltage of 5 kV (30,000× magnification). The scale bar represents 600 nm.

The morphology of MDR *A. baumannii* cells after treatment with phage vWUPSU at an MOI of 1, sacha inchi oil at 0.5× MIC, and the combination of phage vWUPSU and sacha inchi oil was evaluated under SEM. MDR *A. baumannii* without phage vWUPSU or sacha inchi oil treatments was used as a control. SEM revealed that the surface of untreated MDR *A. baumannii* was undamaged and intact (Figure 9a). MDR *A. baumannii* treated with only phage vWUPSU at an MOI of 1 showed cell shrinkage, pore formation, and wrinkled cell surfaces, resulting in cell lysis (Figure 9b). Sacha inchi oil at 0.5× MIC slightly affected MDR *A. baumannii* cells compared with untreated MDR *A. baumannii* and treatment with only phage vWUPSU (Figure 9c). Wrinkled bacterial cell surfaces and small holes were observed. Treatment with the combination of phage vWUPSU and sacha inchi oil yielded more remarkable morphological and structural changes than treatment with phage vWUPSU alone or sacha inchi oil alone (Figure 9d). The results indicated that the combination of phage vWUPSU and sacha inchi oil was more effective than phage vWUPSU or sacha inchi oil treatments alone.

### 2.15. Antibiofilm Activity of the Combination of Phage vWUPSU and Sacha Inchi Oil

**Figure 10:**
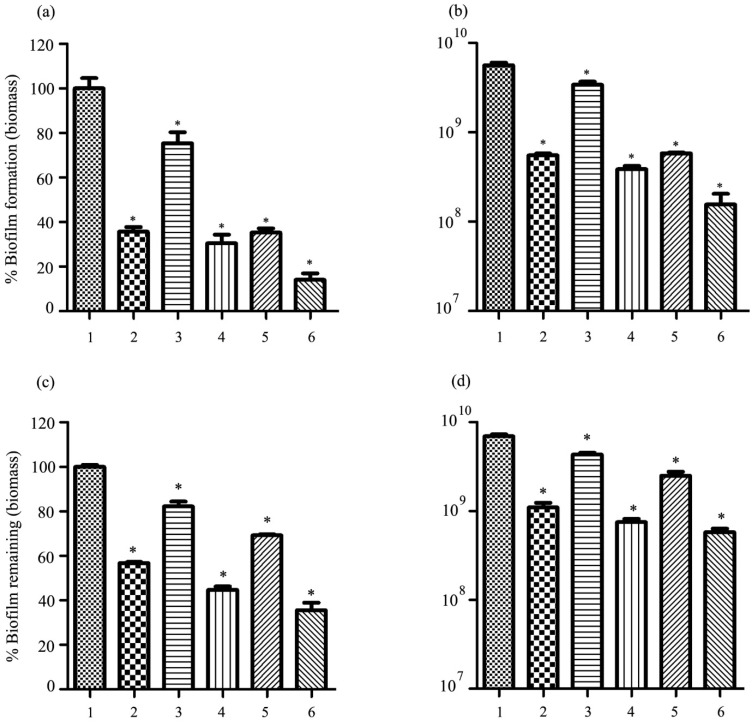
Antibiofilm activity of the combination of phage vWUPSU and sacha inchi oil. The efficacy of the combined phage vWUPSU and sacha inchi oil to reduce biofilm formation was evaluated. (**a**) Biofilm’s biomass and (**b**) bacterial cell viability were assessed. The effect of the combination of phage vWUPSU and sacha inchi oil to remove preformed biofilm was studied. (**c**) Biofilm’s biomass and (**d**) bacterial cell viability were quantitated. Experiments were undertaken independently in triplicate with duplicate assay. The data show mean ± SD (*, *p* value < 0.05). 1 = MDR *A. baumannii*; 2 = MDR *A. baumannii* infected with phage vWUPSU; 3 = MDR *A. baumannii* treated with sacha inchi oil at 0.5× MIC; 4 = MDR *A. baumannii* treated with the combined phage vWUPSU and sacha inchi oil at 0.5× MIC; 5 = MDR *A. baumannii* treated with sacha inchi oil at 1× MIC; 6 = MDR *A. baumannii* treated with the combined phage vWUPSU and sacha inchi oil at 1× MIC.

The combination of phage vWUPSU and sacha inchi oil with regard to the prevention of biofilm formation and destruction of preformed biofilms was investigated. With regard to biofilm formation, the combination of phage vWUPSU at an MOI of 1 and sacha inchi oil at 0.5× MIC and the combined phage vWUPSU at an MOI of 1 and sacha inchi oil at 1× MIC were used to assess the inhibition of biofilm formation in parallel with only phage vWUPSU, only sacha inchi oil, and no treatments. Only phage vWUPSU at an MOI of 1 and only sacha inchi oil at 0.5× MIC to 1× MIC significantly reduced the biofilm’s biomass by approximately 64.3% and 24.7 to 64.7%, respectively (*p* ≤ 0.04) (Figure 10a). Biofilm cell viability significantly decreased by 1.1 log after treatment with only phage vWUPSU at an MOI of 1 (*p* = 0.01) (Figure 10b). For only sacha inchi oil at 0.5–1× MIC treatment, biofilm cell viability significantly reduced by 0.2–1.2 log (*p* ≤ 0.03). A significant reduction in biofilm biomass by approximately 69.5–85.9% was observed after incubation with the combination of phage vWUPSU and sacha inchi oil (*p* ≤ 0.004). Biofilm cell viability was significantly reduced by 1.2–1.4 log (*p* ≤ 0.01). With regard to preformed biofilm destruction, preformed biofilm significantly reduced by approximately 43.7% and 17.8 to 30.8% after adding phage vWUPSU at an MOI of 1 and sacha inchi oil at 0.5× MIC to 1× MIC, respectively (*p* ≤ 0.01) (Figure 10c). The biomass of preformed biofilm treated with the combination of phage vWUPSU at an MOI of 1 and only sacha inchi oil at 0.5× MIC and the combination of phage vWUPSU at an MOI of 1 and only sacha inchi oil at 1× MIC treatments significantly decreased by 55.3% and 64.4%, respectively (*p* ≤ 0.001). After treatment with phage vWUPSU at an MOI of 1 and sacha inchi oil at 0.5× MIC to 1× MIC, significant reductions of 1.6 log and 0.3 log to 0.5 log in viable bacterial cells were detected, respectively (*p* ≤ 0.01). With regard to the combination of phage vWUPSU and sacha inchi oil at 0.5× MIC or 1× MIC, biofilm cell viability significantly reduced by 1.0 log and 1.1 log, respectively, compared with that of the untreated control (*p* ≤ 0.002) (Figure 10d). The combination of phage vWUPSU and sacha inchi oil showed an additive effect (FIC index 0.6) against biofilms.

**Figure 11:**
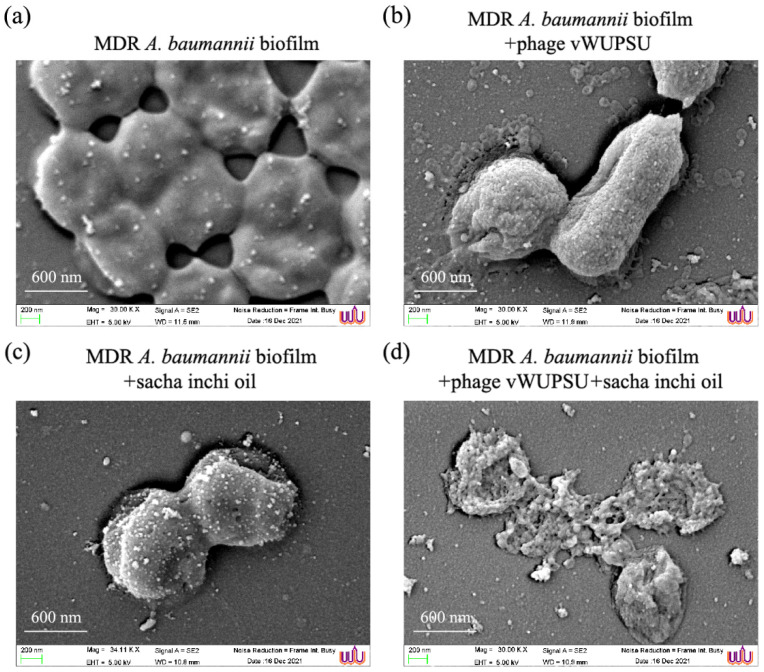
Ultrastructural analysis of MDR *A. baumannii* biofilm after treatment with the combination of phage vWUPSU and sacha inchi oil. The biofilms of MDR *A. baumannii* cells were formed and then treated with (**a**) only TSB, (**b**) phage vWUPSU at an MOI of 1, (**c**) sacha inchi oil at 0.5× MIC, and (**d**) the combined phage vWUPSU and sacha inchi oil. The cells were observed under a field emission scanning electron microscope at an acceleration voltage of 5 kV (30,000× magnification). The scale bar represents 600 nm.

The effects of the combination of phage vWUPSU at an MOI of 1 and sacha inchi oil at a 0.5× MIC on biofilm cells were also confirmed by SEM analysis. SEM revealed undamaged smooth cell surfaces of MDR *A. baumannii* cells under biofilm conditions (Figure 11a). The numbers of MDR *A. baumannii* cells after treatment with only phage vWUPSU and only sacha inchi oil were reduced compared with the untreated condition. MDR *A. baumannii* treated with the phage showed distorted and broken cell morphology, while some cells had small structural alterations (Figure 11b). MDR *A. baumannii* cells treated with sacha inchi oil at a 0.5× MIC showed slight effects such as rough bacterial cell surfaces, pore formation, and cell shrinkage (Figure 11c). Micrographs of the combined phage vWUPSU at an MOI of 1 and sacha inchi oil-treated MDR *A. baumannii* at a 0.5× MIC showed more significant effects on cell morphology compared to phage-only or sacha inchi oil-only treatment. Complete cell lysis was observed (Figure 11d). The results confirmed that biofilm removal by the combination of phage vWUPSU and sacha inchi oil was more effective than by phage vWUPSU alone and by sacha inchi oil treatment alone.

## 3. Discussion

MDR *A. baumannii* is one of the most important pathogens associated with hospital-acquired infections worldwide [35,36,37]. MDR *A. baumannii* causes a wide range of severe infections, such as bloodstream infections, pneumonia, meningitis, wound infections, and urinary tract infections [38]. Recently, MDR *A. baumannii* has developed resistance to most available antibiotics, including carbapenems, colistin, and tigecycline, the last-resort antibiotics available for MDR *A. baumannii* treatment [39,40]. Thus, there is an urgent need to develop and understand new antibiotics and other potential alternatives to prevent infections including, for example, natural products, peptides, enzymes, nanoparticles, and phages. Due to their specificity of action, phages have garnered interest for development as one of the most promising alternatives to antibiotics. Moreover, the US Food and Drug Administration (FDA) approved the use of phages in food applications [41]. For example, ListShieldTM was approved. Phage cocktail can be used to spray RTE meat and poultry to control *Listeria monocytogenes* [42]. Phage products are commercially available against foodborne pathogenic bacteria, including *Escherichia coli* O157:H7, *Salmonella* spp., and *L. monocytogenes* [43]. This work focused on the isolation and characterization of a phage specifically infecting MDR *A. baumannii.* The antibacterial and antibiofilm activities of the combination of this new phage and sacha inchi oil were assessed.

Phage vWUPSU, which was isolated from hospital wastewater samples, exhibited a broad host range. Further investigation on the ability of phage vWUPSU to lyse human symbiotic bacteria should be performed. It is important to show that the phage has minimum safety concerns when clinically applied. The morphological features of phage vWUPSU were observed under SEM analysis, and the whole genome of phage vWUPSU was studied. The results indicated that phage vWUPSU was a novel lytic phage that belonged to the order *Caudovirales*, *Myoviridae* family, and genus *Obolenskvirus*. Based on the phage genome, there were no antibiotic resistance genes, virulence genes, prophage-related genes, and toxin genes. The results indicated that phage vWUPSU was safe for use as a treatment. Biological characterizations of phage vWUPSU were conducted. Phage vWUPSU has a high adsorption rate and a large burst size. Even though phage vWUPSU was sensitive to UV radiation, similarly to other phages [44,45,46], the vWUPSU phage was stable over a wide range of pH values and temperatures. Due to the different pH values of various organs in the human body, the stability of phage vWUPSU at a wide pH has attracted interest in applying for oral administration [47]. The high stability of phage vWUPSU might display good antibacterial activity in the human body. The lytic activity of phage vWUPSU showed that phage decreased the rate of bacterial growth in a dose-dependent manner. The results suggested that phage vWUPSU possessed high antibacterial efficiency. With regard to its plaque morphology, phage vWUPSU formed clear plaques with a halo, which is associated with the phage depolymerase enzyme [15]. The depolymerase enzyme has been reported to have antibiofilm activity that can degrade capsular polysaccharides and EPS. Phage vWUPSU might possess antibiofilm activity; therefore, the antibiofilm activity of phage vWUPSU was assessed. Phage vWUPSU exhibited strong activity with regard to biofilm formation and removing preformed biofilms, indicating that phage vWUPSU is suitable for development as a biocontrol treatment. However, some concerns regarding phage therapy, such as the emergence of phage-resistant bacteria, have been reported [48]. This limitation can be overcome by combination treatments [49,50]. Based on our results, the re-growth of MDR *A. baumannii* after phage vWUPSU treatment was also observed. To avoid this limitation, combined therapy was investigated.

Natural products and their derivatives are interesting as potential alternative candidates for the treatment of bacterial infections. Due to the increasing frequency of infections with antibiotic-resistant bacteria, natural products and their derivatives have become increasing prominent as possibly powerful therapeutics against drug-resistant bacteria [51]. Several natural oils have been shown to function as antibacterial oils such as coconut oil [52], virgin coconut oil [53], peanut oil, and ozonized sunflower oil [54]. Previous studies have shown that a combination of phages (PNO5 and PNO9) and carvacrol could deactivate both planktonic *Pseudomonas syringae* pv. actinidiae and biofilm cells [55]. However, there have been no reports about the antibacterial effect of sacha inchi oil on *A. baumannii.* Sacha inchi oil is extracted from sacha inchi (*Plukenetia volubilis*) seeds, which contain high levels of polyunsaturated fatty acids. Sacha inchi oil has been recognized for its positive antioxidant benefit [56,57]. Moreover, sacha inchi oil has moisturizing and anti-irritation activities [21]. Populations in the Amazon rainforest, which is the origin of sacha inchi plants, routinely apply sacha inchi oil to the skin. The chemical compositions of sacha inchi oil have been reported. Sacha inchi oil contains high levels of fatty acids, flavanoids, tocopherols, sterols, triterpene, and aliphatic alcohols [58]. Interestingly, the effect of sacha inchi oil to reduce *S. aureus* adherence on human skin explants and keratinocytes in vitro has been reported [23]. Thus, sacha inchi oil is an interesting oil for developing a composition of external medicines based on this knowledge. External factors such as the chemical composition of the solution, ions, and pH affect the viability of phage [59]. The sensitivity of phage vWUPSU to sacha inchi oil was investigated. Phage vWUPSU showed the ability to survive in sacha inchi oil, indicating that sacha inchi oil did not affect the stability of phage vWUPSU. Sacha inchi oil might be a good synergistic candidate for development as a medical treatment tool incorporating key phages. The efficiency of sacha inchi oil against MDR *A. baumannii* was investigated. Bacterial growth was inhibited by treatment with only sacha inchi oil at 25% (*v*/*v*), but the combination of phage vWUPSU at an MOI of 1 and sacha inchi oil at 12.5% (*v*/*v*) was able to inhibit bacterial growth and the viable bacterial cells. These results indicate that the combination of phage vWUPSU with sacha inchi oil was able to control the emergence of phage-resistant MDR *A. baumannii*. However, the concentration of sacha inchi oil showing the synergetic effect does not seem to be possible to use for the treatment of internal bacterial infections. The combination of phage vWUPSU and sacha inchi oil might be applied for external use. Even though the most commonly identified sites of *A. baumannii* infection include blood, urinary tract, and pneumonia, skin, and soft tissue infections are increasingly being reported [60,61]. Moreover, the synergistic effect of phage vWUPSU and sacha inchi oil on biofilms was also evaluated. The combinations of phage vWUPSU with sacha inchi oil inhibited biofilm formation and reduced preformed biofilms. The results suggested that the combination of phage vWUPSU and sacha inchi oil probably exerted synergistic and additive effects on planktonic and biofilm cells, indicating the potential use of the combination for the control of MDR *A. baumannii* infections and biofilms. However, the specific functions of sacha inchi oil are still unclear. A previous study reported that sacha inchi oil could prevent *S. aureus* attachment to keratinocytes in vitro [23]. With regard to the compositions of sacha inchi oil, the antibacterial properties of the compounds as found in sacha inchi oil have been reported. Fatty acids are commonly known to possess antimicrobial activities [62]. Flavanoids could reduce both biofilm formation and virulence of *A. baumannii* [63]. Tocopherol showed excellent antibacterial activity against both Gram-positive and Gram-negative bacteria [64]. Sterols exerted antimicrobial effects against Gram-positive bacteria [65]. Triterpenes, one major composition of sacha inchi oil, are known to display significant antimicrobial properties [66]. In previous studies on the antibacterial activity of other oils, pure coconut oil could disrupt the bactaerial cell walls, resulting in bacterial cell dysfunctions. Pure coconut oil also activated the phagocytic capability of the immune cells [53]. Moreover, some chemical compositions of pure coconut oil, such as fatty acids, sterol, topopherol, and triterpene, are similar to the components of sacha inchi oil. Thus, sacha inchi oil may provide an antimicrobial effect by disrupting bacterial cell membranes and inhibiting the efflux pump, resulting in bacterial cell death. However, the percentages of chemical compositions of pure coconut oil and sacha inchi oil are different. The mechanism of each oil depends on the components and combinations of natural products each oil contains. Thus, further studies on the mechanism of sacha inchi oil might improve knowledge and increase the possibility of using sacha inchi oil as a component of new products. Due to the antibiofilm activity of sacha inchi oil, the results indicated that sacha inchi oil also could reduce the biofilm, contributing to a virulence factor. Applying sacha inchi oil as an external use product might activate the immune system of the skin to combat bacterial infections.

In summary, phage vWUPSU, which is specific against MDR *A. baumannii*, was isolated and characterized. A synergistic antimicrobial effect of the interaction between phage vWUPSU and sacha inchi oil on planktonic cells was observed. The combination of phage vWUPSU and sacha inchi oil showed an additive effect against biofilms. The results indicated the potential to use both antimicrobials together as antibacterial and antibiofilm agents. The results obtained in this study shed light on an option to apply phage with natural oils. 

## 4. Materials and Methods

### 4.1. Bacterial Strains and Growth Conditions

MDR clinical isolates of *A. baumannii*, *E. coli*, *K. pneumoniae*, MRSA, and *P. aeruginosa* were provided by the Natural Product Research Center of Excellence, Prince of Songkla University, Songkhla Province, Thailand, and all bacterial isolates were isolated from samples of routine laboratory services at Songklanagarind Hospital, Songkhla Province, Thailand. Bacteria were cultured on tryptic soy agar (TSA, Becton, Dickinson and Company, Franklin Lakes, NJ, USA) at 37 °C for 24 h. Single colonies were picked from a TSA plate and resuspended in 3 mL of sterile tryptic soy broth (TSB, Becton, Dickinson and Company, Franklin Lakes, NJ, USA). Broth was incubated under aerobic conditions at 37 °C with shaking at 150 rpm until reaching log phase or stationary phase.

### 4.2. Isolation of Phage

Wastewater samples were collected from Thasala Hospital, Nakhon Si Thammarat for phage isolation. MDR *A.* *baumannii* NPRCOE 160519 was used as a host strain. Samples were centrifuged to remove debris at 6400× *g* for 15 min at 4 °C and then filtered through a syringe sterile filter 0.22 μm (GVS, Los Angeles, CA, USA). MDR *A. baumannii* was cultured and adjusted to a final OD600 of 0.1 (1 × 10^8^ CFU/mL). Ten milliliters of water sample was supplemented with 10 mL of TSB and then inoculated with 200 μL of MDR *A. baumannii*. The mixture was incubated overnight at 37 °C with continuous shaking at 200 rpm. After centrifugation to remove the debris and bacterial cells at 6400× *g* for 15 min, the supernatant was collected and filtered using a 0.22 μm syringe filter. The presence of phages in the supernatant was examined by the conventional double-layer agar method.

### 4.3. Conventional Double-Layer Agar Method

MDR *A. baumannii* was cultured until reaching log phase or stationary phase. MDR *A. baumannii* was adjusted to a final OD600 of 0.1. The phage was serially diluted in SM buffer (100 mM NaCl, 8 mM MgSO_4.7_H_2_O, and 50 mM Tris-HCl (1 M, pH 7.5)). Two hundred microliters of serially diluted phage solution was mixed with 200 μL of log phase MDR *A. baumannii*. The mixture of MDR *A. baumannii* and phage suspension was added to soft top agar. Subsequently, the mixture was quickly poured onto the TSA plate and permitted to solidify. Plates were incubated overnight at 37 °C.

### 4.4. Phage Purification

The phage was purified by a conventional double-layer agar method as described above. A single plaque was picked by pushing the tip through the overlay agarose and then soaked in 500 μL sterile SM buffer followed by incubation overnight at 4 °C. The serial dilution of the phage was prepared in SM buffer. Two hundred microliters of each dilution of the phage was inoculated into 200 μL of log phase MDR *A. baumannii* clinical isolate. The mixture was mixed with top agar and quickly poured onto the TSA plate. The plate was incubated overnight at 37 °C. Single plaque purification was repeated three times to obtain purified phages.

### 4.5. Phage Stock Preparation

A conventional double-layer agar method was used to prepare phage stock as previously described [46,67]. Briefly, the phage was serially diluted, and 200 μL of the diluted phage was mixed with 200 μL of log phase *A. baumannii* clinical isolate [68]. The mixture was added in top agar and then quickly poured onto the TSA plate. After incubation overnight at 37 °C, semiconfluent plates were selected for phage elution. The phage was eluted by adding 5 mL of SM buffer and incubating at 4 °C for 6 h. The supernatant was collected, pooled, and centrifuged to remove bacterial cells at 6400× *g* for 20 min at 4 °C. Subsequently, the supernatant was collected, filtered through a sterile 0.45 µm cellulose acetate filter, and kept as a phage stock at 4 °C until used.

### 4.6. Determination of Phage Titer

Phage titer was determined by a conventional double-layer agar method. A series of ten-fold dilutions of the phage was prepared in SM buffer. Two hundred microliters of the phage was mixed with 200 μL of log-phase MDR *A. baumannii* followed by conventional double-layer agar method. The plates were incubated overnight at 37 °C. The plaques were counted and then calculated as a plaque forming unit per milliliter (PFU/mL).

### 4.7. Phage Morphology under Transmission Electron Microscopy (TEM)

TEM was performed to observe the morphology of phage particles by negative staining technique [46,69]. Twenty microliters of the phage solution was dropped onto a copper grid followed by staining with 2% (*v*/*v*) uranyl acetate. The grid was gently touched with respect to the filter paper for removing excess liquid and dried. The phage particles were observed under a JEOL JEM-2010 transmission electron microscope at an acceleration voltage of 160 kV.

### 4.8. Phage Host Range Testing

The host range of the phage was evaluated by spot testing of the phage on a bacterial lawn [70]. Briefly, two hundred microliters of each log phase MDR *A. baumannii* clinical isolate was mixed with soft top agar and then quickly poured onto the TSA plate. After solidifying, a ten microliter aliquot of the phage (10^5^ PFU/mL) was plated onto the overlaid top agar. The plates were incubated overnight at 37 °C to observe the clear zone in a bacterial lawn due to bacterial lysis by the phage. The experiment was undertaken independently in duplicate with duplicate spot testing.

### 4.9. EOP

All MDR *A. baumannii* clinical isolates sensitive to the phage in the spot test were further evaluated to determine the EOP value, as previously described [46]. Briefly, each MDR *A. baumannii* isolate was cultured until the log phase was reached and then adjusted to a final OD600 of 0.1. The phage was serially diluted in SM buffer. Two hundred microliters of each dilution of the phage was mixed with 200 µL of each MDR *A. baumannii* isolate followed by incubation at room temperature for 15 min. The lysis ability was assessed by the double-agar overlay method. The EOP was calculated as the ratio of the average PFU on target bacteria to the average PFU on host bacteria. EOP was classified as highly productive (EOP ≥ 0.5), medium productive (0.1 ≤ EOP < 0.5), low productive (0.001 < EOP < 0.1), or insufficient (EOP ≤ 0.001) [25]. The experiment was undertaken independently in duplicate with a duplicate plaque assay.

### 4.10. Whole Genome Analysis

Whole genome sequencing was performed commercially on the Illumina sequencing platform (Macrogen Inc., Seoul, South Korea). The DNA of phage vWUPSU was extracted from the phage stock, and genomic DNA quality was assessed. The genome library was prepared following the instructions for the TruSeq Nano DNA library prep kits. Briefly, DNA fragments were converted into the library by ligation to 5′- and 3′-adapters followed by PCR amplification of the libraries. DNA was purified by gel purification and then sequenced. Quality control of the raw reads was performed, and the low-quality ends were trimmed. Filtered reads were assembled de novo as contigs by various k-mers using the SPAdes assembler [71]. The genome was annotated by Prokka [68]. Protein coding sequences, tRNA genes, and rRNA genes were analyzed. For functional analysis, all protein-coding genes were blasted in the NCBI database. The RAST server was also used to annotate the genome [26]. Virulence genes in the genome were searched by VirulenceFinder 2.0 [72]. The tRNA genes in genomic sequences were also searched using the tRNAscan-SE web server [73]. Circular representations of the genome were constructed by the CGview [28].

The genetic relationship of phage vWUPSU was investigated by BLASTN, and the phylogentic tree of the whole-phage genome was constructed with the VICTOR Classification and Tree Building Online Resource server [30]. The nucleotide sequences were compared by the Genome-BLAST Distance Phylogeny (GBDP) method using default setting [74]. Intergenomic distances were used to analyze the balanced minimum evolution tree with branch support via FASTME including SPR postprocessing [75]. Branch support was inferred from 100 pseudobootstrap replicates each. FigTree was used to visualize the phylogenetic tree and rooted at the midpoint. The taxon boundaries at the species, genus, and family levels were assessed by the OPTSIL program with the recommended clustering thresholds and an F value of 0.5 [30,76,77]. The ORFs from phage vWUPSU and the closest phage were compared by the VipTree server [27].

### 4.11. Phylogenetic Tree of Genes

The phage terminase large subunit and endolysin annotated from the RAST server were selected as models for evaluating the genetic relationships. The phylogenetic trees based on the alignment of amino acid sequences were constructed by maximum-likelihood phylogenetic tree based on the JTT matrix-based model. The similarity between sequences of phage vWUPSU and other phages in the NCBI database was compared by BLASTX. The sequences of relative phages using a maximum E value of 0.00 and a minimum identity of 60% were retrieved from the database. The maximum likelihood phylogenetic trees using 1000 bootstrap replicates were constructed in MEGA-X.

### 4.12. Phage Adsorption Rate Assay

The adsorption assay was undertaken as described elsewhere [69]. Briefly, MDR *A. baumannii* was cultured until reaching the log phase and then incubated with phage vWUPSU at an MOI of 1. The mixture was incubated at 37 °C with constant shaking and collected every 5 min post incubation for 30 min. The samples were centrifuged, and the supernatant was collected for filtration through a 0.22 μm pore filter. The filtrate was serially diluted in SM buffer and then plated by a double-layer plaque assay for plaque counting. The experiments were undertaken independently in duplicate with duplicate plaque assay.

### 4.13. One Step Growth Curve

A one step growth curve of phage vWUPSU was carried out as described previously, using a MOI of 0.001 with phage vWUPSU [78]. Briefly, MDR *A. baumannii* was cultured until reaching log phase (ca. OD600 ~ 0.5). Phage vWUPSU was diluted in SM buffer to 5 × 10^6^ PFU/mL. One hundred microliters of phage vWUPSU was added to a tube with 9.9 mL MDR *A. baumannii* culture. The tube was swirled gently in an incubator at 37 °C for 5 min. One hundred microliters of the mixture was transferred to a tube with 9.9 mL of fresh prewarmed TSB as tube A. The mixture was mixed well, and one milliliter of the mixture was then transferred to tube B with 9 mL of TSB. The mixture was mixed well, and one milliliter of the mixture was then transferred to tube C with 9 mL of TSB. Samples were incubated at 37 °C and then taken at 5 min intervals until 90 min. At each time point, samples were collected and then filtered through a sterile 0.22 µm cellulose acetate filter. The soft-agar overlay method was performed. Plate counts were plotted to obtain the one-step growth curve. The latent period and burst size were calculated. The experiments were undertaken independently in duplicate with a duplicate plaque assay.

### 4.14. Bacterial Cell Killing Assay

The lytic efficiency of phage vWUPSU was determined by OD600 measurements. MDR *A. baumannii* was cultured overnight and then adjusted to a final OD600 of 0.1. MDR *A. baumannii* was infected with phage vWUPSU at MOIs of 0.1, 1, 10, and 100. The mixtures were incubated at 37 °C with shaking at 150 rpm and then collected for OD600 measurements every hour for 8 h. Uninfected MDR *A. baumannii* was used as a control. The experiments were undertaken independently in duplicate with a duplicate assay.

### 4.15. TEM Study of the Morphology of Phage-Infected Bacteria

TEM was used to visualize the effects of phage vWUPSU on MDR *A. baumannii* cells following the methods in a previous report [46]. Briefly, MDR *A. baumannii* was infected with phage vWUPSU at an MOI of 1 for 3 h. The samples were centrifuged and washed two times with 0.1 M PBS (pH 7.4) followed by fixation in 2.5% glutaraldehyde/PBS overnight at 4 °C. After washing with PBS, the samples were incubated for 2 h with 1% (*w*/*v*) osmium tetroxide prepared in PBS. The fixed samples were processed through an ethyl alcohol dehydration series and Embed 812 resin infiltration. The ultrathin sample sections were obtained using an ultramicrotome and then mounted on nickel grids. Ultrathin sections were stained on the grid with uranyl acetate and lead citrate. The sample images were taken under a JEOL JEM-2010 transmission electron microscope at an acceleration voltage of 160 kV.

### 4.16. Assessment of Phage Stability under Thermal and pHs

The effects of temperature and pH on the stability of phage vWUPSU were determined. For thermal stability testing, phage vWUPSU was diluted to a final concentration of 10^8^ PFU/mL in a final volume of 1 mL of SM buffer for testing at 4 °C to 80 °C. Phage samples for testing at −80 °C and −20 °C were diluted in SM buffers supplemented with glycerol to a final glycerol concentration of 40%. The samples were incubated at specific temperatures (−80 °C, −20 °C, 4 °C, 25 °C, 37 °C, 40 °C, 50 °C, 60 °C, 70 °C, and 80 °C). After 2 h post incubation, phage samples at different temperatures were cooled slowly and then placed in an ice–water bath. To test the effects of pH on phage stability, phage vWUPSU was diluted in SM buffer at different pH values (pH 1 to pH 14) followed by incubation for 2 h. The phage suspensions at different pH values were neutralized to pH 7. The stability of phage vWUPSU under different temperatures and pH conditions was determined by titration using a conventional double-layer agar method. Phage stability at 25 °C and pH 7 served as controls. The experiments were undertaken independently in duplicate with a duplicate plaque assay.

### 4.17. Impact of UV Radiation on Phage Stability

Stability of phage vWUPSU under UV radiation was assessed following the methods in previous reports [45,46]. Briefly, phage vWUPSU was diluted to a final concentration of 10^8^ PFU/mL in a final volume of 10 mL of SM buffer. The phage suspension was added to open Petri dishes. The disks containing the phage sample were incubated on ice and positioned at a distance of 30 cm from the UV-C light source. The samples exposed to the light were collected every 10 min for 1 h and the titer was determined by a conventional double-layer agar method. The experiments were undertaken independently in duplicate with a duplicate plaque assay.

### 4.18. Antibiofilm Activities of Phage vWUPSU

The effectiveness of phage vWUPSU at preventing biofilm formation and removing biofilms was assessed by using the method described previously [46,69,79]. Briefly, log-phase MDR *A. baumannii* was diluted in TSB to an OD600 of 0.1. To determine the activity of phage vWUPSU with regard to preventing biofilm formation, 100 µL of MDR *A. baumannii* was added to 96-well plates and then supplemented with 100 µL of phage vWUPSU at 1 × 10^1^ to 1 × 10^8^ PFU/well. The plates were incubated at 37 °C without agitation for 24 h. To determine the activity of phage with regard to the removal of preformed biofilms, 100 µL of MDR *A. baumannii* was added to 96-wells plates and cultured at 37 °C overnight. The supernatant was removed, and the wells were then washed with PBS. Phage vWUPSU at 1 × 10^1^ to 1 × 10^8^ PFU/well was added to the wells, and the plates were incubated under standard conditions for 24 h. To determine biofilm biomass and biofilm cell viability, the supernatant was discarded, and then two washes with PBS were performed. The biofilm biomass was assessed by adding 0.1% crystal violet (Sigma-Aldrich Chemicals, St. Louis, MO, USA). After incubation for 30 min, the supernatant was removed, and two washes with PBS were performed. The plates were air dried, and the biofilm’s biomass was solubilized by adding 200 µL of 95% ethanol followed by measurement at 600 nm. To quantify viable biofilm cells, the supernatant was discarded, and then two washes with PBS were performed. Sterile 0.9% NaCl was added to the wells, and the biofilms were suspended. Serial dilutions were performed in 0.9% NaCl, and 10 µL of diluted supernatant was plated on TSB by the microdrop technique. The plates were incubated at 37 °C overnight, and the colonies were counted, followed by CFU/mL determination.

### 4.19. Evaluation of Phage Sensitivity to Sacha Inchi Oil

The effects of sacha inchi oil on phage vWUPSU viability were examined. Briefly, sacha inchi oil was purchased from Chiangrai Agriculture Development Co., Ltd. (Chiangrai, Thailand) [24]. Working sacha inchi oil was prepared by dilution in PBS. Phage vWUPSU was incubated with 1% (*v*/*v*) to 90% (*v*/*v*) sacha inchi oil diluted in PBS at 37 °C. Phage vWUPSU without sacha inchi oil was used as a negative control. Phage viability was examined at 24 h post incubation using the double-layer agar method. The experiments were undertaken independently in duplicate with a duplicate plaque assay.

### 4.20. Determination of the MIC and MBC Values of Sacha Inchi Oil against MDR A. baumannii

Sacha inchi oil was evaluated against MDR *A. baumannii* to determine MIC and MBC. This assay was performed in 96-well microtiter plates using a modified broth microdilution method described in the guidelines of the Clinical and Laboratory Standards Institute [80]. Briefly, MDR *A. baumannii* was cultured at 37 °C with agitation at 120 rpm until bacterial growth reached log-phase growth. The bacterial culture was adjusted in Mueller Hinton Broth (MHB) to OD600 of 0.1, approximately 1 × 10^8^ CFU/mL, and then diluted in MHB to 1 × 10^6^ CFU/mL. Sacha inchi oil was added to the 96-well plates and then serially diluted two-fold in MHB. One hundred microliters of MDR *A. baumannii* was added to the wells containing 50 µL of the serial two-fold dilution of sacha inchi oil in MHB. Fifty microliters of MHB was supplemented to the wells and gently mixed. The plates were incubated at 37 °C for 18 h. Bacterial viability was detected by adding resazurin (Sigma-Aldrich Chemicals, St. Louis, MO, USA). The lowest concentration before the color change was selected to determine MIC. To determine MBC, 10 µL of each concentration was plated onto MHA plates followed by incubation at 37 °C overnight. The concentration at which bacteria did not grow was the MBC value. This experiment was undertaken independently in triplicate with duplicate.

### 4.21. Combination of Sacha Inchi Oil and Phage vWUPSU for Bactericidal Assay

Phage vWUPSU was combined with sacha inchi oil to inhibit the growth of MDR *A. baumannii* to evaluate the possibility of developing a medical application. Four different treatments were evaluated: control (no treatment); only phage (an MOI of 1); only sacha inchi oil (1 × −0.02× MIC); and the combination of phage (an MOI of 1) and sacha inchi oil (1 × −0.02× MIC). Briefly, MDR *A. baumannii* was cultured at 37 °C for 6 h to reach the mid-log growth phase. The bacterial suspension was adjusted to a final OD600 of 0.1 and then diluted to 1 × 10^6^ CFU/mL. One hundred microliters of bacterial suspension was added to the plate, which contained 50 μL of 1 × −0.02× MIC of sacha inchi oil in MHB or phage vWUPSU diluted in MHB. Fifty microliters of MHB was supplemented to the wells and gently mixed. The combined effect of sacha inchi oil and phage vWUPSU was assessed using the concentrations assessed for phage vWUPSU or sacha inchi oil alone. The plates were incubated at 37 °C without agitation for 18 h, and MIC and MBC tests were evaluated using the protocol described above.

### 4.22. Killing Kinetics of the Antibacterial Activity of Phage vWUPSU and Sacha Inchi Oil Combination

The killing kinetics of the combined phage vWUPSU and sacha inchi oil were evaluated using 96-well plates. MDR *A. baumannii* was cultured at 37 °C overnight. The bacterial suspension was adjusted to a final OD600 of 0.1 and then diluted to 1 × 10^6^ CFU/mL. Sacha inchi oil was serially diluted two-fold in MHB, and 50 µL of sacha inchi oil at 0.5× MIC was added to 96-well plates, after which 50 µL of phage vWUPSU was added at an MOI of 1. Only sacha inchi oil at 0.5× MIC and only phage vWUPSU at an MOI of 1 were assessed in parallel as controls. The plates were incubated at 37 °C without agitation, and bacterial growth was assessed by measuring the optical density at 600 nm every hour for 10 h. After incubation at 37 °C for 24 h, bacterial growth was determined by measuring optical density at 600 nm, and the viability of the bacteria was assessed by counting the viable bacterial cells. The bacterial suspension was serially diluted in a sterile 0.9% NaCl solution, and the number of bacteria was determined by counting colony-forming units on TSA plates. This experiment was undertaken independently in triplicate with a duplicate assay.

### 4.23. Effects of Combined Phage vWUPSU and Sacha Inchi Oil on Bacterial Morphology under Scanning Electron Microscopy (SEM)

The effects of phage vWUPSU and sacha inchi oil on MDR *A. baumannii* were visualized by SEM analysis. Briefly, MDR *A. baumannii* was cultured at 37 °C overnight and then diluted to a final OD600 of 0.1. MDR *A. baumannii* was treated with a combination of phage vWUPSU at an MOI of 1 and sacha inchi oil at 0.5× MIC. MDR *A. baumannii* was treated with only phage vWUPSU at an MOI of 1 or only sacha inchi oil at 0.5× MIC in parallel. MDR *A. baumannii* with no treatment was used as a control. After incubation at 37 °C for 3 h, the samples were centrifuged at 6000× *g*, and the supernatant was discarded. Bacterial pellets were washed twice with PBS followed by fixation. For SEM analysis, MDR *A. baumannii* cells were fixed with 2.5% glutaraldehyde in 0.1 M PBS for 2 h and then washed with 0.1 M sodium phosphate buffer. After removing the supernatant, the cells were fixed with 1% OsO_4_ in DI water and then washed with DI water. The cells were dehydrated in a series of ethanol solutions (20%, 40%, 60%, 80%, 90%, and 100%) and then dried by a critical point dyer technique. The samples were coated with gold and then observed under a field emission scanning electron microscope (Merlin compact, Zeiss, EDX (Oxford, Aztec, Oxford, UK), EBSD (Oxford, Nordlys Max, Oxford, UK)).

### 4.24. Antibiofilm Activity of Phage vWUPSU and Sacha Inchi Oil Combination

The effectiveness of the combined phage vWUPSU and sacha inchi oil with regard to controlling MDR *A. baumannii* biofilm formation and inactivating preformed biofilms was evaluated. To assess the effect on biofilm formation, MDR *A. baumannii* was cultured at 37 °C overnight and then diluted to a final OD600 of 0.1. One hundred microliters of MDR *A. baumannii* was added to 96-well plates. Sacha inchi oil was serially diluted two-fold in MHB, and 50 µL of sacha inchi oil at 0.5–1× MIC was added to 96-well plates containing MDR *A. baumannii*. Fifty microliters of phage vWUPSU at an MOI of 1 was then added. The effects of using only sacha inchi oil at 0.5–1× MIC and only phage vWUPSU at an MOI of 1 were assessed in parallel as controls. The plates were incubated at 37 °C for 24 h, and the effectiveness of the combined phage vWUPSU and sacha inchi oil was measured. To assess the ability of the combination of phage and sacha inchi oil to remove preformed biofilms, the number of viable biofilm cells was measured. MDR *A. baumannii* was cultured and then diluted. One hundred microliters of MDR *A. baumannii* was added to 96-well plates and then incubated at 37 °C for 24 h to allow biofilm formation. At 24 h post-incubation, the supernatant was discarded, and then two washes with PBS were performed. Sterile 0.9% NaCl was added to the wells, and the biofilms were suspended. Serial dilutions were performed in 0.9% NaCl, and 10 µL of diluted supernatant was plated on TSB by the microdrop technique. The plates were incubated at 37 °C overnight and the colonies were counted followed by CFU/mL determination. For testing the ability of the combination of phage and sacha inchi oil to remove preformed biofilms, MDR *A. baumannii* was cultured in 96-well plates and then incubated to allow biofilm formation. At 24 h post-incubation, the supernatant was gently removed, and the wells were washed twice with PBS. Sacha inchi oil was serially diluted two-fold in MHB, and 50 µL of sacha inchi oil at 0.5–1× MIC was added to 96-well plates. Fifty microliters of phage vWUPSU at an MOI of 1 was then added. Only sacha inchi oil at 0.5–1× MIC and only phage vWUPSU at an MOI of 1 were assessed in parallel as controls. Subsequently, the plates were incubated at 37 °C. At 24 h post-infection, the supernatant was removed, and then two washes with PBS were performed. The biofilm biomass was assessed by adding 0.1% crystal violet. After incubation for 30 min, the supernatant was removed and then washed twice with PBS. The plates were air dried, and 200 µL of 95% ethanol was added to solubilize the biofilm’s biomass followed by measurements at 600 nm. To quantify the viable biofilm cells, the supernatant was discarded, and then two washes with PBS were performed. Two hundred microliters of 0.9% NaCl was added to the wells, and the biofilms were suspended. Serial dilutions were performed in 0.9% NaCl, and 10 µL of diluted supernatant was plated on TSB by the microdrop technique. The plates were incubated at 37 °C overnight, and the colonies were counted. CFU was then determined. The minimum biofilm inhibition concentration 50% (MBIC_50_) was selected to calculate the FIC index. The FIC index was interpreted according to the following: synergistic (∑FIC: ≤0.5), partial synergy (∑FIC: >0.5 but <1), additative (∑FIC: >0.5 and ≤1), indifferent (∑FIC: >1 and ≤4), and antagonistic (∑FIC: >4) [34].

### 4.25. SEM Analysis of Biofilms

The antibiofilm activity of phage vWUPSU and sacha inchi oil combination against MDR *A. baumannii* cells was visualized under SEM. Briefly, the effectiveness of the combination of phage vWUPSU at an MOI of 1 and sacha inchi oil at 0.5× MIC on biofilm formation was selected as a model. MDR *A. baumannii* was mixed with the combination of phage vWUPSU at an MOI of 1 and sacha inchi oil at 0.5× MIC. MDR *A. baumannii* cells were allowed to form biofilms on glass slides. Only phage vWUPSU at an MOI of 1, only sacha inchi oil at 0.5× MIC, and no treatment were performed in parallel. At 24 h post-incubation, the samples were fixed following the fixation protocol for SEM analysis as described above.

### 4.26. Statistical Analyses

The data were analyzed by the GraphPad Prism program (GrapPad Software). Statistical analysis of significance was undertaken by unpaired *t*-test using SPSS, *p* < 0.05 for significance.

### 4.27. Accession Number

The phage genome sequence was submitted to GenBank under accession number OL743187.

## 5. Conclusions

These findings may lead to the development of complementary and alternative treatments to combat antimicrobial resistance in MDR *A. baumannii*. Moreover, the antimicrobial mechanism involved in the activity of sacha inchi oil and especially the combination of phage vWUPSU together with sacha inchi oil should be further investigated. This study highlights the search for innovative therapeutical approaches and options for combining antibiotics, phages, and (other) naturally occurring factors in the combat against antibiotic resistance, including options for fruitful cooperation between various expert centers in these areas.

## Figures and Tables

**Table 1:** Host range infection and EOP of phage vWUPSU.

Strain	Phage vWUPSU	
	Lytic Activity	EOP
MDR *A.* *baumannii* NPRCOE 160516	−	−
MDR *A.* *baumannii* NPRCOE 160517	+	Low productive (0.08)
MDR *A.* *baumannii* NPRCOE 160518	−	−
MDR *A.* *baumannii* NPRCOE 160519	+	High (Host = 1)
MDR *A.* *baumannii* NPRCOE 160520	−	−
MDR *A.* *baumannii* NPRCOE 160521	+	Medium productive (0.25)
MDR *A.* *baumannii* NPRCOE 160522	−	−
MDR *A.* *baumannii* NPRCOE 160523	−	−
MDR *A.* *baumannii* NPRCOE 160524	+	Medium productive (0.43)
MDR *A.* *baumannii* NPRCOE 160525	−	−
MDR *A.* *baumannii* NPRCOE 160526	+	High productive (0.63)
MDR *A.* *baumannii* NPRCOE 160527	+	Low productive(0.09)
MDR *A.* *baumannii* NPRCOE 160528	+	High productive (0.75)
MDR *A.* *baumannii* NPRCOE 160529	+	Medium productive (0.29)
MDR *A.* *baumannii* NPRCOE 160530	−	−
MDR *A.* *baumannii* NPRCOE 160531	+	High productive (0.77)
MDR *A.* *baumannii* NPRCOE 160532	+	Low productive(0.04)
MDR *A.* *baumannii* NPRCOE 160533	−	−
MDR *A.* *baumannii* NPRCOE 160534	−	−
MDR *A.* *baumannii* NPRCOE 160535	−	−
MDR *A.* *baumannii* NPRCOE 160536	+	High productive (0.73)
MDR *A.* *baumannii* NPRCOE 160537	+	Medium productive (0.1)
MDR *A.* *baumannii* NPRCOE 160538	−	−
MDR *A.* *baumannii* NPRCOE 160539	−	−
MDR *A.* *baumannii* NPRCOE 160540	+	Medium productive (0.14)
MDR *A.* *baumannii* NPRCOE 160541	+	High productive (0.63)
MDR *A.* *baumannii* NPRCOE 160542	+	High productive (0.84)
MDR *A.* *baumannii* NPRCOE 160543	−	−
MDR *A.* *baumannii* NPRCOE 160544	+	Medium productive (0.11)
MDR *A.* *baumannii* NPRCOE 160545	−	−
*E. coli*	−	−
*K. pneumoniae*	−	−
MRSA	−	−
*P. aeruginosa*	−	−

+ was able to produce lytic zone, − was unable to produce lytic zone. The EOP was classified as highly productive (EOP ≥ 0.5), medium productive (0.1 ≤ EOP < 0.5), low productive (0.001 < EOP < 0.1), or insufficient (EOP ≤ 0.001) [25].

## Data Availability

Data sharing not applicable.

## Supplementary Materials

The following supporting information can be downloaded at: https://www.mdpi.com/article/10.3390/ph15030291/s1, Table S1: Annotation of the putative functional ORFs in phage vWUPSU genome was analyzed by BLASTX, Table S2: Annotation of the putative functional ORFs in phage vWUPSU genome was conducted using the RAST database.
